# Visual gene-network analysis reveals the cancer gene co-expression in human endometrial cancer

**DOI:** 10.1186/1471-2164-15-300

**Published:** 2014-04-23

**Authors:** Wei-Chun Chou, An-Lin Cheng, Marco Brotto, Chun-Yu Chuang

**Affiliations:** 1 Department of Biomedical Engineering and Environmental Sciences, National Tsing Hua University, Hsinchu 30013, Taiwan; 2 Muscle Biology Research Group-MUBIG, Schools of Nursing & Health Studies, University of Missouri Kansas City, Kansas City, MO 64110, USA

**Keywords:** Endometrial cancer, WGCNA, Network analysis, Hub gene, TCA cycle

## Abstract

**Background:**

Endometrial cancers (ECs) are the most common form of gynecologic malignancy. Recent studies have reported that ECs reveal distinct markers for molecular pathogenesis, which in turn is linked to the various histological types of ECs. To understand further the molecular events contributing to ECs and endometrial tumorigenesis in general, a more precise identification of cancer-associated molecules and signaling networks would be useful for the detection and monitoring of malignancy, improving clinical cancer therapy, and personalization of treatments.

**Results:**

ECs-specific gene co-expression networks were constructed by differential expression analysis and weighted gene co-expression network analysis (WGCNA). Important pathways and putative cancer hub genes contribution to tumorigenesis of ECs were identified. An elastic-net regularized classification model was built using the cancer hub gene signatures to predict the phenotypic characteristics of ECs. The 19 cancer hub gene signatures had high predictive power to distinguish among three key principal features of ECs: grade, type, and stage. Intriguingly, these hub gene networks seem to contribute to ECs progression and malignancy via cell-cycle regulation, antigen processing and the citric acid (TCA) cycle.

**Conclusions:**

The results of this study provide a powerful biomarker discovery platform to better understand the progression of ECs and to uncover potential therapeutic targets in the treatment of ECs. This information might lead to improved monitoring of ECs and resulting improvement of treatment of ECs, the 4^th^ most common of cancer in women.

## Background

Endometrial cancers (ECs) are the most common type of uterine cancer. With more than 280,000 cases occurring annually worldwide, it has become the fourth most common cancer in women worldwide [1]. The incidence rate of ECs is higher than uterine cervix cancer and ovarian cancer in the United State [2]. These tumors have been broadly classified into two major subtypes I and II, based on the clinic-pathological characteristics such as prognosis and aggressiveness, as well as, molecular alterations that impact tumor response to therapies [3]. Type I tumors are the most frequent subtype which have been linked to obesity, estrogen excess, and low-grade (differentiated) inflammation. Type II endometrial tumors are serous carcinoma that have been associated with older, non-obese, post-menopausal women, high-grade (undifferentiated), and also with worst outcomes. It has been suggested that the molecular-genetic alterations may be responsible for the distinct morphology and biologic behavior of the different subtypes of human ECs [4]. For example, low-grade or early-stage Type I tumors may progress to high-grade or late-stage; nonetheless Type I and II cancers appear to be separate entities in most cases, and different molecular abnormalities would result in unique cellular functions and distinctive tumor morphology [5]. Therefore, it is crucial to discover with more accuracy the putative molecular signatures of ECs, which should allow for improved detection and monitoring of endometrial tumorigenesis, since such knowledge could be beneficial for early diagnostic, enhanced prognostic, and more effective therapeutic strategies.

ECs are characterized by a variety of genetic alterations and significant gene expression modifications. Over the last decade, multiple studies have identified aberrant gene expression of several important genes in ECs, with the mutation frequency varying according to the histological classification [6]. Type I tumors are frequently characterized by the loss or altered expression of phosphatase and tensin homolog (*PTEN*). *PTEN* modulates cell survival and proliferation through its effects on downstream factors, mainly phospholipid phosphatidylinositol (3, 4, 5)-triphosphate (*PIP3*) and protein kinase B (*PKB*, *Akt*). *PTEN* inactivation leads to a decrease of lipid and protein phosphatase activity and promotes cell cycle progression to the G1/S phase [7]. Other genes are linked to abnormalities in Type I tumors includingβ-catenin, *K-ras* and DNA-mismatch repair genes [7-10]. In comparison, Type II tumors have been reported to be associated with abnormalities in *TP53* and *Her2/neu*[6]. The gene *TP53* encodes a tumor suppressor *p53*, the most frequently mutated protein in cancer. P53 prevents cell cycle progression after DNA damage, inducing cell arrest and apoptosis through several regulator proteins such as *p21*, *Cyclin D1*, and *RB1. TP53* mutations occur as an early event in Type II tumorigenesis and may occur as manifestations of late-stage molecular changes in Type I lesions. Overexpression of *Her-2/neu* observed in Type II carcinomas has been linked to coding alterations for a transmembrane receptor tyrosine kinase involved in cell signaling [11]. Although these studies provide important insights into the molecular basis of endometrial cancers, a limited set of well-known cancer genes was obtained from these studies. In fact, until now, a large-scale screen of the gene expression analyses incorporating systematic methods to discover cancer subtypes and their molecular alterations in ECs has not been globally conducted and explored.

Recent advances in constructing genetic network approaches have enabled the unprecedented characterization of studying a variety of somatic alterations and gene expression in cancer genomes. Therefore, these advances allow connecting the existent gap of understanding the association of individual genes to complex diseases such as cancer by the systematic investigation of the observed relationship between gene products and tumorigenesis. A weighted gene co-expression network approach (WGCNA) has been proposed to reconstruct gene co-expression networks (modules) in terms of large-scale gene expression profiles and as well as for the distinction of centrally located genes (hub genes) driving key cellular signaling pathways [12,13]. The WGCNA approach provides a functional interpretation in Systems Biology and leads to new insights into cancer pathophysiology [14-17].

Here, we aimed to establish a systematic framework for constructing for the first time, the ECs-associated gene co-expression networks and pin-pointing cancer hub genes contributing to endometrial tumorigenesis and progression. This study provides a novel and broad application platform for the identification of cancer gene signatures of ECs tumorigensis and for the discovery of potential new molecular targets for the development of more effective therapies for the treatment of ECs.

## Results

### Systematic framework for identifying cancer hub genes in ECs

**Figure 1:**
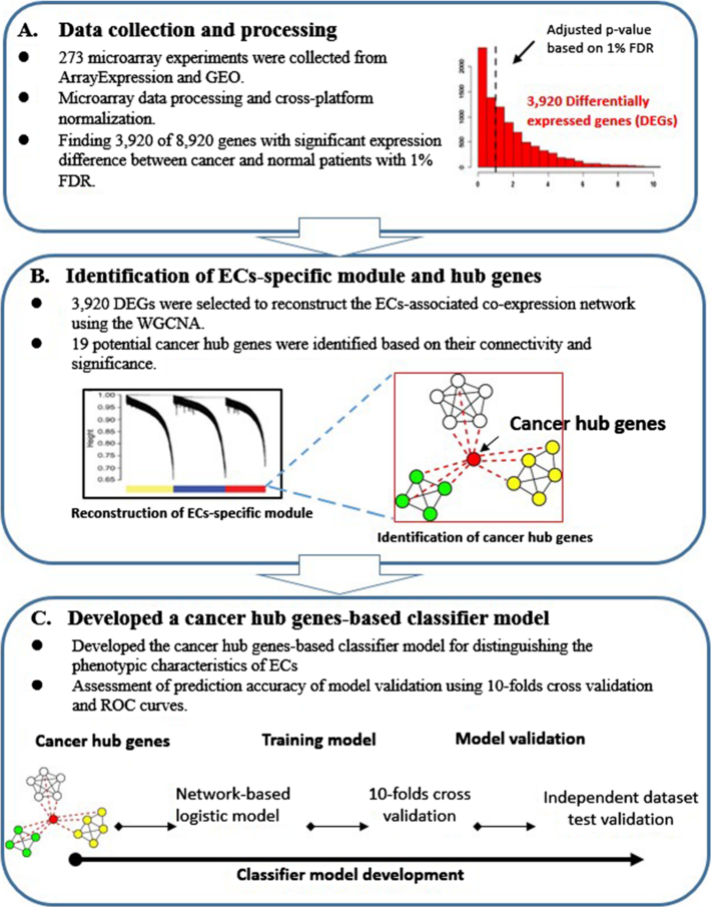
Framework for deriving the cancer hub genes and validating its phenotypic relevance in ECs: (A) Data collection and processing for 273 microarray experiments to find 3,920 DEGs, (B) Identification of ECs-specific module and 19 cancer hub genes, and (C) Developed a cancer hub genes-based classifier model using 10-fold cross validation and ROC curves to assess the prediction accuracy of model.

In this study, a novel systematic analysis was developed to integrate WGCNA and elastic-net analysis to identify cancer hub genes in ECs (Figure 1). This method is divided into three main parts. First, we performed a very large human microarray-based ECs meta-analysis by merging multiple platforms to reveal the differentially expressed genes on 273 EC samples properly matched with normal samples. Second, we conducted the WGCNA analysis to reconstruct EC-associated gene co-expression networks (modules) and discover the cancer hub genes. Third, we developed a cancer hub genes-based classifier model to distinguish the phenotypic characteristics of ECs (i.e., grade, type and stage). Finally, we used the hub genes as the gene signatures to validate its biological and phenotypic characteristics relevance using 10-fold cross-validation and independent data set validation. Figure 1 presents an overview of approach in this study, and the detailed methods are described in Supporting Information (Additional file 1: SI Materials and Methods).

### Reconstruction of EC-specific gene co-expression network

**Figure 2:**
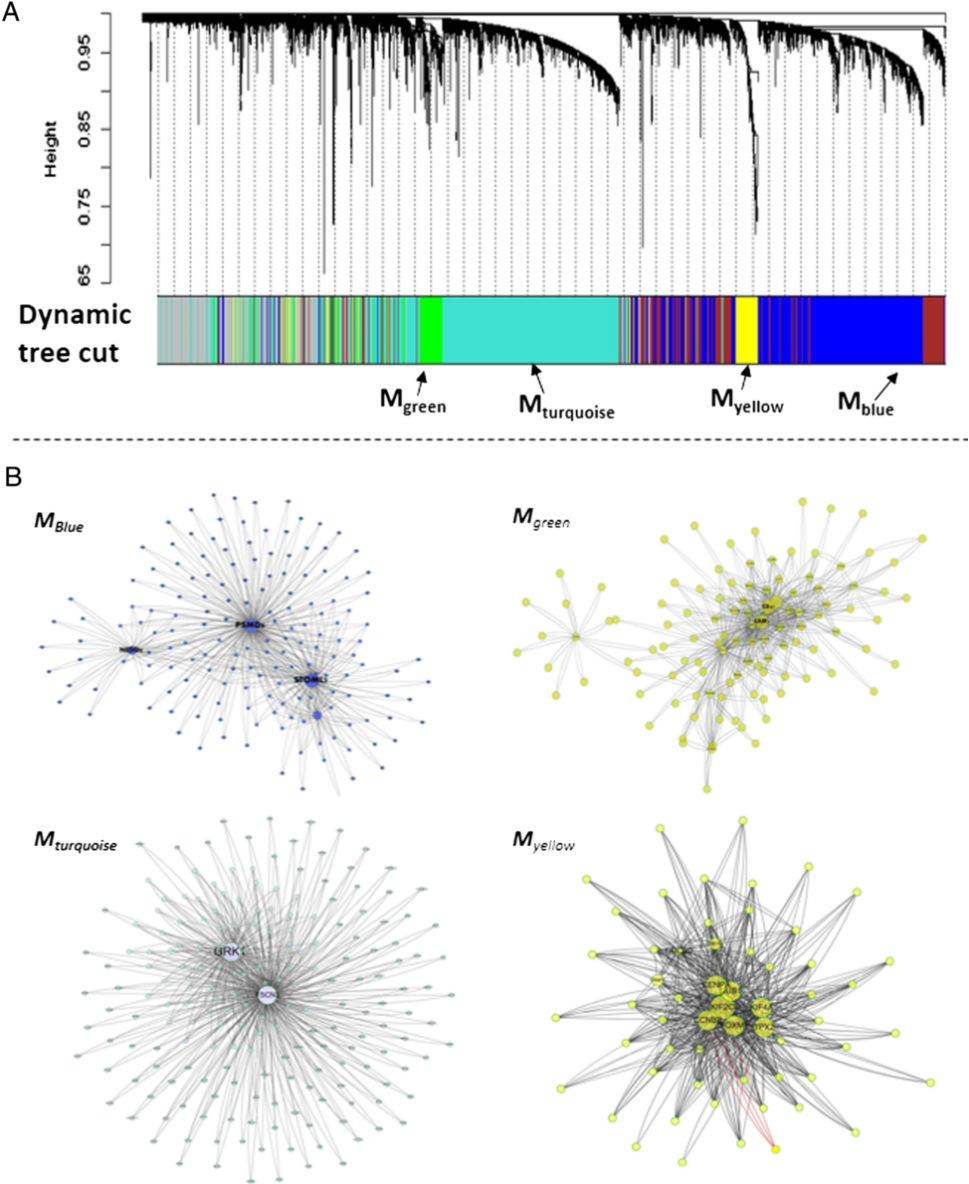
**WGCNA analysis on the large-scale microarray datasets. (A)** Dendrogram showing relationship for the topological overlap of genes and their relationship to modules, which are color-coded. **(B)** Graphic depiction of the blue-color module (M_blue_), green-color module (M_green_), turquoise-color module (M_turquoise_) and yellow-color module (M_yellow_) using Cytoscape. For each viewing module, pairs of genes with the highest intramodular topological overlap are illustrated, with each link corresponding to a topology overlap measure (TOM) between the connected nodes.

In the initial analysis, we identified 3,920 genes having significant expression difference between subjects with cancer and subjects without cancer by applying a 1% FDR (Additional file 2: Table S1). These endometrial cancer-related genes were used to reconstruct the EC-associated co-expression network (module) and identify a number of modules of high co-expression genes. As shown in Figure 2, these modules are significantly enriched for biologically important processes that are relevant to cancer, including cell-cycle regulation, antigen processing, immune response, and cell adhesion (Table 1). Among the modules, yellow-colored module (M_yellow_) that specifically corresponds to clinical information of ECs, showed a high Pearson correlation with phenotypic characteristics of ECs including grade (*r* = 0.44, Bonferroni-adjusted *p-value* = 1.2E^−16^), type (*r* = 0.34, Bonferroni-adjusted *p-value* = 6.3E^−9^) and stage (*r* = 0.31, Bonferroni-adjusted *p-value* = 2.1E^−7^) in ECs. The blue-colored module (M_blue_) was only significantly correlated with the stage of ECs (*r* = 0.42, Bonferroni-adjusted *p-value* = 6.0E^−19^). By contrast, other modules showed a much lower correlation with the phenotypic characteristics of ECs. Interestingly, the M_yellow_ module was significantly enriched for cell-cycle regulation (Bonferroni-adjusted *p-value* = 1.2E^−31^). Conversely, M_blue_ gene ontology categories included antigen processing (Bonferroni-adjusted *p-value* = 8.7E^−12^) and the citric acid (tricarboxylic acid; TCA) cycle (Bonferroni-adjusted *p-value* = 4.5E^−12^).

**Table 1:** Module significance in ECs and GO analysis

**Module**	**Correlation**^**a **^**with phenotypic characteristics of ECs (p-value)**			**Gene ontology enrichment analysis**		
	**Grade**	**Type**	**Stage**	**Term**	**p-value**^**b**^	**FDR**
Blue	−0.002 (9.0E^−1^)	0.08 (2.2E^−1^)	0.42 (6.0E^−19^)	Antigen processing	8.7E^−12^	1.7E^−12^
				TCA cycle	4.5E^−12^	2.1E^−12^
Green	0.006 (9.1E^−1^)	−0.02 (6.1E^−5^)	0.01 (9.3E^−1^)	Immune response	3.7E^−43^	6.3E^−40^
Turquoise	−0.17 (6.4E^−3^)	−0.19 (2.4E^−3^)	0.17 (6.2E^−3^)	Cell adhesion	2.9E^−29^	1.1E^−29^
Yellow	0.44 (1.2E^−16^)	0.34 (6.3E^−9^)	0.31 (2.1E^−7^)	Cell-cycle regulation	4.9E^−35^	8.6E^−32^

^a^The correlation coefficient was calculated from the module eigengenes (i.e., first principal component of the expression values across subjects) and phenotypic characteristics of ECs using Pearson correlation.

^b^p-value used the Bonferroni-adjusted p-value.

### Identification of cancer hub genes

**Figure 3:**
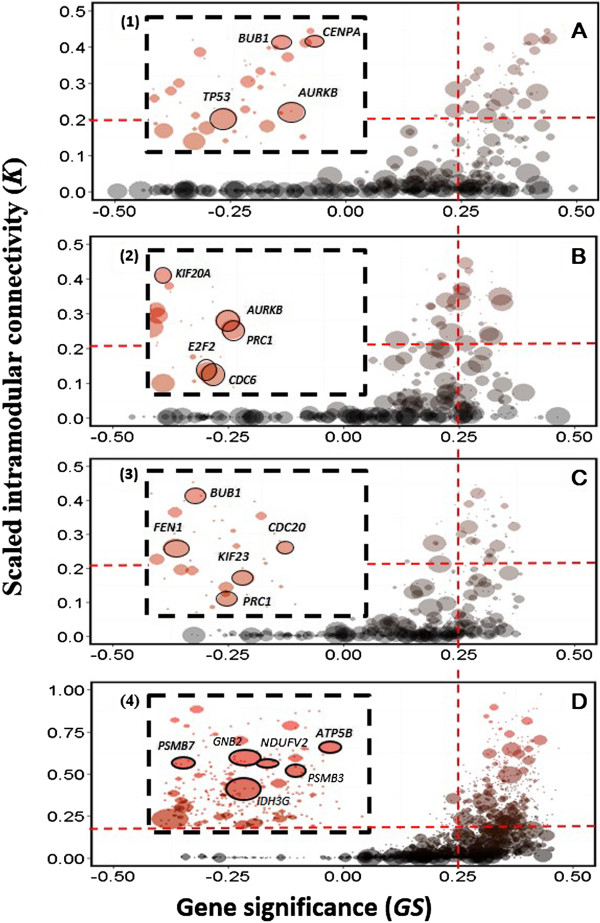
**A systematic screen in ECs-associated networks identifies cancer hub genes.** A plot representation of the elastic-net regression gene selection results showing the Gene significance (GS, x-axis) and scaled gene connectivity (K, y-axis) of all genes of M_yellow_ network that are associated with **(A)** Grade, **(B)** Type, **(C)** Stage and that of the M_blue_ network that are associated with Stage **(D)**. Each circle represents a single phenotype-gene interaction and the size is proportional to the frequency (*f*) calculated from the elastic-net regression analysis. The red dashed line indicates the cancer hub genes selection criteria (GS > 0.25, K > 0.2). Insets (1) - (4) are magnified views of selected cancer hub genes.

**Figure 4:**
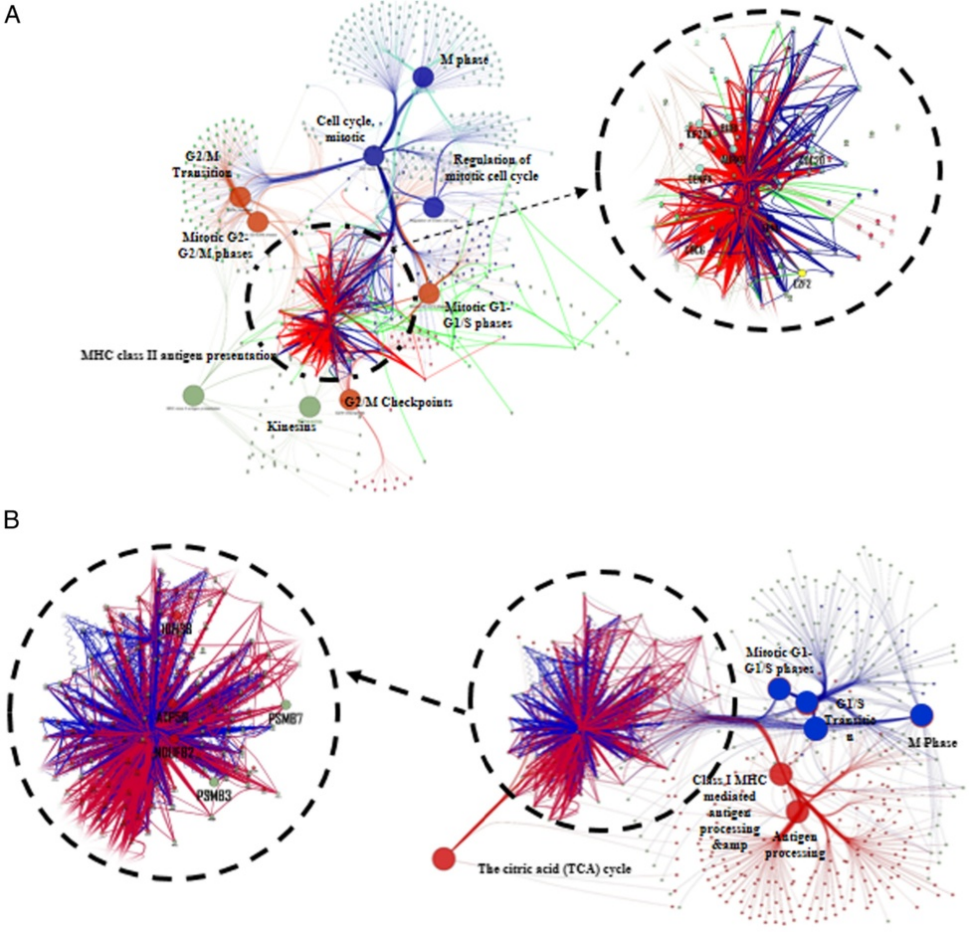
**Differentially activated pathways features between Type I and Type II ECs subtypes in (A) the cell‒cycle regulator network and (B) the antigen processing network.** Color arrows reflect pathway differences between subtypes (blue: Type I, red: Type II). Green lines represent activation. A zoomed in view of the cancer hub gene signatures are also shown.

Genes with the highest degree of connectivity within a module (centrally located genes of co-expressed genes) are termed hub genes and are expected to be drivers required for signaling pathways of essential cellular function. To identify the cancer hub genes in the M_yellow_ and M_blue_ modules, we estimated the scale connectivity (*K*) for each gene and for gene significance (GS) based on its Pearson correlation with phenotypic characteristics of ECs (Additional file 3: Table S2) and predicted the frequency (*f*) by using the elastic net regression model combined with bootstrap approaches in the modules (Figure 3). We set the weighted cutoff value (defined as *r* > 0.2, *K* > 0.25 and *f* > 750) to identify cancer hub genes with strongest connections to other genes and to link to phenotypic characteristics of ECs. As a result, we identified 19 cancer hub genes with at least 50 connections derived from the M_yellow_ and M_blue_ Modules. These hub genes associated with grade (*TP53*, *BUB1*, *AURKB* and *CENPA*), type (*AURKB*, *PRC1*, *CDC6*, *E2F2*, *KIF20A*) and stage (*BUB1*, *FEN1*, *KIF23*, *CDC20* and *PRC1*) in the M_yellow_, module. Nonetheless, 5 hub genes associated with stage including *IDH3G*, *NDUFV2*, *ATP5B*, *PSMB3* and *PSMB7* were identified in the M_blue_ module (Figure 3A- 3D, Table 2). Figure 4 illustrated the relationship among these genes suggesting a complex regulatory gene network with varying topology.

**Table 2:** List of hub gene associated with grade, type and stage o ECs

**Symbol**	**Gene names**	Hub gene significance^*^		
		***f***	***K***	***GS***
**Hub genes for grade**				
*TP53*	Tumor protein 53	954	0.40	0.31
*AURKB*	Aurora kinase B	885	0.41	0.37
*BUB1*	Budding uninhibited by benzimidazoles 1	768	0.43	0.42
*CENPA*	Centromere protein A	764	0.44	0.42
**Hub genes for type**				
*AURKB*	Aurora kinase B	995	0.41	0.35
*PRC1*	Protein regulator of cytokinesis 1	893	0.34	0.33
*CDC6*	Cell division cycle 6	849	0.26	0.24
*E2F2*	E2F transcription factor 2	776	0.31	0.22
*KIF20A*	Kinesin family member 20A	760	0.46	0.21
**Hub genes for stage (derived from module yellow)**				
*BUB1*	Budding uninhibited by benzimidazoles 1	768	0.43	0.42
*FEN1*	Flap structure-specific endonuclease 1	764	0.27	0.32
*KIF23*	Kinesin family member 23	763	0.34	0.27
*CDC20*	Cell division cycle 20	762	0.37	0.32
*PRC1*	Protein regulator of cytokinesis 1	760	0.34	0.27
**Hub genes for stage (derived from module blue)**				
*IDH3G*	Isocitrate dehydrogenase 3 (NAD+) gamma	934	0.36	0.49
*NDUFV2*	NADH dehydrogenase (ubiquinone) flavoprotein 2	873	0.35	0.42
*ATP5B*	ATP synthase, H + transporting, mitochondrial F1 complex, beta polypeptide	769	0.52	0.49
*PSMB3*	Proteasome (prosome, macropain) subunit, beta type, 3	762	0.41	0.47
*PSMB7*	Proteasome (prosome, macropain) subunit, beta type, 7	760	0.32	0.42

*This study defined the hub gene significance (HGS) by integrating WGCNA and elastic-net analysis, estimating the gene significance (*GS*), scaled connectivity (*K*) and frequency (*f*). Only genes present in GS > 0.2, K > 0.2 and f > 750 were selected as ECs-specific hub genes.

### Pathway analysis of hub genes

This study reconstructed and identified the gene networks of cancer hub genes to search the potential key regulators of endometrial tumorigenesis and to identify regulatory relationships among cancer hub genes. Using functional enrichment analysis, we identified three major signaling pathways; cell-cycle regulated networks (mitotic, G2/M or G1/S phases), antigen processing (Class I MHC antigen processing) and the TCA cycle, as the main Gene Ontology biological processes overrepresented in the gene networks deriving from the cancer hub genes (Figure 4). In the cell-cycle network, *BUB1*, *AURKB*, *CENPA*, *KIF20A, CDC20, CDC6, E2F2* and *FEN1* seem to regulate distinct co-expression pathways that contributed to mitotic G2-G2/M phase, G2/M checkpoints, and kinesins in Type II ECs, whereas these genes regulate the M phase and mitotic G1-G1/S phase in Type I ECs (Figure 4A). In addition, the antigen processing and TCA cycle networks are regulated by the hub genes *IDH3G*, *NDUFV2*, *ATP5B, PSMB3* and *PSMB7*, contributing to tumorigenesis in Type II ECs (Figure 4B).

### Cancer hub genes classification based model

**Figure 5:**
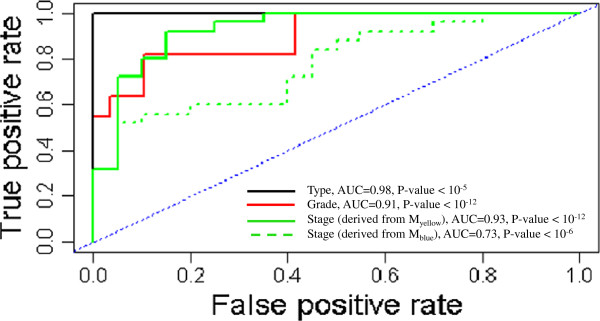
**ROC curve to assess the accuracy of the cancer hub genes signature in the independent dataset.** True positive rate represent the model sensitivity, whereas false positive rate is one minus the specificity or true negative rate and represents chance.

This study performed receiver-operator characteristic curve (ROC) analysis to assess the predictive accuracy of the cancer hub gene signatures. As follows, an AUC (area under curve) value of 0.5 indicated that the predictive performance equals chance, while values greater than 0.5 indicated high predictive capacity. Using a strict 10-fold cross-validation, the classification capacity of cancer hub gene signatures proved to be significantly better than random predictability (Additional file 4: Figure S1A-S1D for second supporting information figure, *p* <10^−6^, AUC = 0.72 ~ 0.85). Furthermore, the module showed a substantial capacity to distinguish ECs grade (AUC = 0.91, *p* < 10^−12^), type (AUC = 0.98, *p* < 10^−5^) and stage (M_yellow_: AUC = 0.93, *p* < 10^−12^; M_blue_: AUC = 0.73, *p* < 10^−4^) in independent datasets (Figure 5). Strikingly, these hub genes deriving from the co-expression networks of M_yellow_ and M_blue_ provided significant predictive power in distinguishing the phenotypic characteristics of ECs.

## Discussion

In this study, we integrated a large-scale transcriptional profiling of 273 ECs datasets to identify appropriate cancer subtypes and biomarkers. Our long-term goal is to provide insights into disease biology and diagnostic classification, which may guide early-phase of clinical therapeutic applications. We also determined that co-expression networks reflect causative relationships between different gene-gene interactions. First, this study found that an EC-specific co-expression network majorly regulated the tumorigenesis of ECs. This network not only enriched genes known to play critical roles in cell cycle regulation but also showed significant correlation with grade, type, and stage of ECs. Second, 19 highly connected hub genes were defined into two EC-associated co-expression networks. Detailed and systematic network analysis revealed that these hub genes played roles as major regulators in cell-cycle regulation, kinase modulation, and the development of various tumors. Finally, for independent analysis of microarray data as validated data sets, these hub genes can provide excellent diagnostic power in distinguishing grade, type, and stage of ECs. Altogether these findings strongly suggest that the role of these hub genes in the EC-associated networks may provide new insights into the underlying biological mechanisms driving the tumorigenesis of ECs.

Tumor cell progression is typically associated with aberrant cell-cycle regulation, and this study identified several unique hub genes in the networks associated with the grade of ECs including *TP53*, *BUB1*, *AURKB, CENPA*. In agreement with previous reports [17-20], the disruption of these hub genes was observed in a variety of human tumors and often correlates with tumorigenesis. For example, *BUB1* has been suggested to play a direct role in the suppression of *p53*-mediated cell death via physical interaction with p53 at kinetochores in response to mitotic spindle damage [18]. Altered expression of *BUB1* is associated with therapy failure and death in patients with multiple types of cancer [19]. Fu et al. (2007) [20] reported a possible mechanism on the roles of aurora kinases in mitosis and tumorigenesis. They found that overexpression of *AURKA* and *AURKB* disruption overrides spindle checkpoint, resulting in the aneuploidy or polyploidy that occurs during cell division. When cells lose the normal function of *p53*, they enter mitosis and become aneuploid, which is considered a cancerous manifestation.

Recently, up-regulation of *BUB1* gene has been linked to the inhibition of p53-dependent senescence, hyper-activation of *AURKB* and phosphorylation of *CENP-A*, and might be considered as a potential oncogene in driving the aneuploidiation and tumorigenesis [21]. Interestingly, this study found *BUB1* not only highly connected with other genes in the co-expression network, but also significantly correlated with the transformation of ECs. Therefore, *BUB1* gene seems to modulate the expression of tens or hundreds of genes suggesting that it might allow undifferentiated cancer cells to overcome apoptotic checkpoints favoring aberrant progression through mitosis.

Previous reports suggested that *PTEN* mutations occur early in the neoplastic process of Type I ECs and co-exist frequently with other mutations in the PI(3)K/AKT pathway [22], yet Type II ECs showed an alteration of the E2F–retinoblastoma protein–p16 pathway by mutations of *p53* and *p16*[23]. In this study, several unique hub genes (*AURKB, PRC1*, *CDC6*, *E2F2*, *KIF20A*) were identified in the co-expression network associated with the subtype of ECs, which were linked to the *PTEN* and *p16*-associated pathway. The cell division gene, *CDC6*, is required for the cell cycle G1-to-S transition. Wu et al. (2009) [24] have identified that *CDC6* is the putative *PTEN* target and demonstrated a causal linkage between *CDC6* and *PTEN* in metastatic human prostate cancer. They indicated that the regulation of *CDC6* expression by *PTEN* is mediated through the *E2F* transcriptional factor and the E2F2 protein. Furthermore, overexpression of *AURKB* has been suggested to compromise the tumor suppressor function of *p53*[25]. The gene *PRC1*, encodes one of the polycomb-group proteins, and is involved in cytokinesis. Of note, the PRC1 and PRC2/3/4 proteins can induce the repression of the *PTEN* transcription via binding to the *PTEN* promoter region in leukemic cells [26], while *KIF20A* is known to be controlled by the E2F–retinoblastoma protein–p16 pathway, and is linked to tumor aggressiveness in human hepatocellular carcinomas [27]. Taken together, it appears that this gene-signature is commonly linked to *PTEN*, *PI(3)K*/*AKT* and E2F–retinoblastoma protein–*p16* pathway, and may be marginally associated with the subtype of ECs.

Cancer stage is the most import indicator for selecting an appropriate cancer treatment option for a patient. In this study, two co-expression networks were significantly correlated with the stage of ECs. These two network-regulated signaling events contributed to cell-cycle regulation, antigen processing and TCA cycle, respectively. From the cell-cycle regulation network, 5 hub genes were identified including *BUB1*, *FEN1*, *KIF23*, *CDC20* and *PRC1. BUB1* and *CDC20* are involved in the M phase of mitotic cell cycle and DNA replication, and play critical roles in the cell-cycle regulation [28]. Altered expression of cell-cycle associated protein like *BUB1* and *CDC20* may lead to chromosomal instability. *BUB1* and *CDC20* are important regulators of the anaphase-promoting complex (APC) [29]. APC’s function is to trigger the transition from metaphase to anaphase by tagging critical cell-cycle proteins for degradation, and maintaining genomic stability in a normal manner. *BUB1* can directly phosphorylate the APC’s co-activator *CDC20*, leading to the decreased activity of APC, which determines the metaphase-to-anaphase transition. The overexpression of *BUB1* or C*DC20* induces misregulation of APC, and is associated with the chromosomal instability and poor outcomes in breast cancer patients [30,31]. *KIF23* belongs to the kinesin family, and it is part of the central spindle in a complex that clusters PRC1 and AURKB together at the spindle midzone to enable anaphase in dividing cells and regulation of central spindle assembly [32]. Abnormal regulation of these genes may lead to the chromosomal instability, thereby promoting tumor development and progression.

The pathway of MHC class I presentation is an important mechanism in determining whether tumors are able to evade immune response. Down-regulation of MHC Class I has been described in ovary and cervix malignancies. Abnormal expression of MHC Class I genes has been linked to the advanced stage of disease and poor survival in ovarian cancer [33]. Recently, a large cohort study of patients with endometrial cancer indicated that down-regulation of MHC Class I expression in endometrial cancer patients are correlated to late-stage ECs [34]. In this study, we identified a group of hub genes associated with the stage of ECs deriving from the MHC Class I co-expression network. These hub genes, *PSMB7* and *PSMB3*, were found to significantly regulate the network of antigen processing and to contribute to Class I MHC mediated processing. Furthermore, we also discovered 3 hub genes (*IDH3G*, *NDUFV2* and *ATP5B*) associated with the TCA cycle. No other study to date has reported that these hub genes might be associated with cancer, but recent studies have indicated that the alterations in the TCA cycle enzymes may favor tumorigenesis by impacting on cellular redox state and overall cell metabolism [35,36]. Therefore, it is reasonable to hypothesize that these hub genes could be involved with tumor progression.

To identify the signaling pathway through which hub genes regulated the co-expression network in the progression of ECs, we compared the gene co-expression networks between Type I (early-stage, low-grade) and Type II ECs (late-stage, high-grade) of ECs. In the cell-cycle regulation network, Type II ECs displayed a distinct pathway when compared with Type I ECs. However, *BUB1*, *AURKB*, *CDC6*, *CENPA* and *KIF20A* seemed to be critical regulators for the co-expression network in Type II compared to Type I ECs. Among these genes, *BUB1* may play a key role in regulating these genes to promote tumor formation. The overexpression of *BUB1* regulates *AURKB*, *CENPA*, *CDC6* and *KIF20A* in a variety of human cancers [37-39]. Furthermore, from the antigen processing and TCA cycle co-expression networks, we found that the network regulated TCA cycle through the co-regulation of *IDH3G*, *NDUFV2* and *ATP5B*, and linked to the pathway of Cass I MHC antigen processing key players were *PSMB3* and *PSMB7* in Type II compared to Type I ECs. Expression of these genes in Type II ECs may facilitate the identification of signaling pathways contributing to tumor progression.

## Conclusions

This study used a novel systematic framework to identify two co-expression networks associated with ECs tumorigenesis based on large-scale human microarray data. In addition, a number of novel hub genes in these two co-expression networks were identified contributing to three signaling pathways: cell-cycle regulation, antigen processing and TCA cycle, and presented a high predictive power in distinguishing grade, type and stage of ECs. Although Type I and Type II ECs shared similar genetic information, several critical hub genes were identified that may contribute to progression of ECs. Together, these findings provided a clearer and broader picture of the signaling pathways regulated by co-expression networks contributing to ECs. Furthermore, the characterization of these hub genes might infuse novel insights into the identification of novel clinical markers and potential therapeutic targets for ECs.

## Methods

### Data collection and processing

Microarray data sets were systematically searched from ArrayExpress (http://www.ebi.ac.uk/arrayexpress/) using the keyword “endometrial cancer”. Only the studies that presented the raw microarray expression data in humans (women) were employed in this study. Samples from both patients with and without cancer were requested along with phenotypic characteristics of ECs such as grade, type, and stage. These samples were controlled in a way that none of the samples had been exposed to any specific treatment, not subjected to any stimulus, nor derived from cell lines, thus only from endometrial tissue of women. A total of 273 microarray datasets from multiple platforms including Affymetrix, Agilent and Illumina were merged across platforms as training datasets to uncover the predictive cancer signatures (Additional file 1: SI Materials and Methods). In addition, 65 samples from Illumina and Swegene microarray platforms were utilized as validation datasets. Therefore, a total of 318 microarray datasets were effectively used in these studies. All the information of the datasets are summarized in Table 3 and Additional file 5: Table S3. To merge the microarray datasets measured with multiple platform chips, we selected genes from all platforms based on the NIH Entrez Gene ID and used the Cross-Platform Normalization (XPN) method of Shabalin et al. [40] implemented in the R package: “CONOR” [41]. These normalization procedures led to a total of 8,920 genes that were selected to further analysis after data processing. Detailed descriptions of the data preprocessing and normalization are summarized in Additional file 6: Figure S2 and Additional file 1: SI Materials and Methods.

**Table 3:** Characteristics of microarray datasets for platform, sample groups, and grade, type and stage in endometrial cancers

**Characteristics**	**Training dataset (n = 273)**	**Validation set A (n = 40)**	**Validation set B (n = 90)**
**Sample**			
Control	37 (14%)	20 (50%)	45 (50%)
Case	236 (86%)	20 (50%)	45 (50%)
**Grade**^**a**^			
G1	86 (36%)		20 (44%)
G2	43 (18%)	-	12 (27%)
G3	52 (22%)	-	8 (18%)
Unknown	55 (23%)	-	5 (11%)
**Type**^**a**^			
T1	113 (48%)	10 (50%)	-
T2	84 (36%)	10 (50%)	-
Unknown	39 (17%)	0	-
**Stage**^**a**^			
IA	31 (13%)	10 (50%)	10 (22%)
IB	37 (16%)	-	10 (22%)
IC or late stage	23 (10%)	10 (50%)	19 (42%)
Unknown	82 (61%)	0	6 (14%)
**Platform**			
Affymetrix	140	-	-
Illumina	19	20	-
Agilent	114	-	-
Swegene	-	-	45

^a^Endometrial cancers are grouped by the grade (degree of differentiation), type (histopathological types) and stage (status of spread).

### Initial data analysis

We reviewed the sample profiles in each of the 273 microarray datasets. From these 273 datasets, at least four samples corresponding to both classes of one analysis of interest were selected for additional analyses. Thus, analyses of interest included cancer versus non-cancer patients, cancer grade, which was further divided into high grade (G3, poorly differentiated) versus low grade (G1, well differentiated), cancer types; Type I (estrogen dependent) versus Type II (estrogen independent), and cancer stage (higher than stage 2) versus early stage (lower than stage 2). After the assignment of samples to classes, we assessed the differential expression using Student’s *t* test to identify the significantly differential expression of gene profiles. False discovery rates (FDR) were used in these analyses for correcting for multiple comparisons [42]. All differentially expressed analysis used the “*limma*” program in the R-based Bioconductor package to calculate the level of differential expression [43].

### Reconstruction of co-expression network

This study performed WGCNA analysis to construct the modules of co-expression gene for the EC-associated networks and their interactions. From the processed expression files, the networks were formed from the weighted correlation matrices following the protocols of WGCNA. Briefly, the WGCNA converts the gene expression profiles into connection weights that can be visualized as topology overlap measures (TOM) (Additional file 7: Figure S3). We chose expression profiles of 4,500 genes in the co-expression network analysis. These genes were either significantly differentially expressed between non-cancer and cancer samples (FDR < 0.05 and fold change > 1.5 between two groups) or showed a large variability in expression. We defined modules using a hierarchical cluster method, and used the topological overlap dissimilarity measure (1-TOM) as the distance measure with a height cutoff value of 0.95 and a minimum size (gene groups) cutoff value of 100 for the resulting dendrogram. All network analyses were implemented in the package WGCNA in the R environment as previously described [44].

### Identification of cancer hub genes

We implemented for the first time an unique systematic framework that applies the elastic-net regularization-based approach and WGCNA to take the ECs-specific gene co-expression networks into account in the process of identification of cancer hub genes. Our approach contain two major steps. Firstly, we used WGCNA to identify the cancer hub genes that functionally contribute to the tumorigenesis of ECs. To distinguish centrally located genes (hub genes) of the co-expressed network, we calculated its scaled connectivity (*K*) and genes significance (*GS*) using WGCNA. This approach enabled us to determine the hub genes implicated by both the genetic marker and network connectivity information.

Secondly, to link these potential hub genes to the phenotypic characteristics of ECs, the elastic-net analysis was used to select which of these features were significantly associated with phenotypic characteristics of ECs across the gene co-expression networks. This approach is ideal for building the linear models in situations where the number of variables significantly outweighs the number of samples. In fact, this approach has been used as a powerful classification algorithm for large-scale microarray analysis [45,46]. In addition to all these innovative but highly structured and systematized approaches and procedures, we alsoperformed bootstrap analysis, sampling the datasets with replacements 1,000 times, and calculating the frequency (*f*) of markers (genes) for inclusion in the model for each bootstrap sample. Only genes present in more than the fourth quartile (*f >* 750) of all bootstrap samples were selected as ECs-specific hub genes. All elastic-net analysis used the R package “glmnet” [47]. Full methods are available in Support information.

### Classifier predictive model

Classification performance was assessed with areas under the receiving operating characteristic (AUC) curve. Using the penalized logistic regression via the elastic-net, a classification model was built, and its discriminatory capacity was first estimated with a strict 10-fold cross-validation methodology (as described in Additional file 1: Materials and Methods). The resulting model was next tested on independent datasets using the cancer hub genes as a model input to predict the classes of particular samples relevant to the process of neoplastic transformation and progression in ECs.

### Pathway analysis

The network was visualized through Cytoscape Software 3.0.1 [48]. This study used the Cluepedia plug-in in Cytoscape to identify potential association to pathways of cancer hub genes [49]. CluePedia organizes a functionally grouped pathway with cancer hub genes by integrating heterogeneous expression data and functional network information.

## Competing interests

All the authors have declared that no competing interests exist.

## Authors’ contributions

Conceived and designed the analysis: WCC CYC. Planned statistical design: WCC CYC ALC MB. Analyzed the data: WCC CYC ALC. Wrote the paper: WCC CYC ALC MB. All authors read and approved the final manuscript.

## Supplementary Material

Additional file 1 SI Materials and Methods. Click here for file

Additional file 2: Table S1 Genes differentially expressed between tissues of cancer and non-cancer samples subjects. Click here for file

Additional file 3: Table S2 List of potential hub genes in the WGCNA network, their gene significance and connectivity to each module. Click here for file

Additional file 4: Figure S1 10-fold cross validation to predict the (A) grade, (B) type, (C, D) stage of ECs using grade-, type- and stage-related cancer hub genes. The ROC curve of stage II is predicted from the cancer hub genes derived from M_blue_. Click here for file

Additional file 5: Table S3 List of microarray experiments used for meta-analysis. Click here for file

Additional file 6: Figure S2 Detailed process of matching probes among different microarray platforms. Click here for file

Additional file 7: Figure S3 Topological overlap matrix (TOM) plot of the network connections. Genes in the rows and columns are sorted by the clustering tree. Click here for file
