# Supplementary material for: Visual gene-network analysis reveals the cancer gene co-expression in human endometrial cancer
Source: BMC Genomics. 2014 Apr 23;15:300. doi: 10.1186/1471-2164-15-300 (PMC4234489; doi:10.1186/1471-2164-15-300)
Supplement: Additional file 1 — SI Materials and Methods. [file 1471-2164-15-300-S1.docx]

Supporting Information

SI Materials and Methods

1. Data set collection

Microarray data sets were systematicallysearched from GEO (<http://www.ncbi.nlm.nih.gov/geo/>) and ArrayExpress (<http://www.ebi.ac.uk/arrayexpress/>) using the keyword “endometrial cancer”. Only the studies that presented the raw microarray expression data were employed in this study. Samples from both normal and cancer patients were requested along with clinical classifier information such as grade, type, and stage. These samples were control, non-treatment, not subjected to any stimulus, nor derived from cell lines.A total of 273 human microarray data sets from multiple platforms including Affymetrix (HG-U133 Plus2.0, n=186 and HG-U133A), Agilent (Agilent Whole Human Genome Oligo Microarray (G4112F)) and Illumina (Illumina Human-6 v2 Expression BeadChip) were cross-platform merged as training data sets to uncover the predictive signatures. The 65 samples from two microarray data sets (Illumina HumanHT-12 v3.0 Expression BeadChip, n=40) and Swegene microarray platforms (SWEGENE_BAC_32K_Full, n=90) were utilized as validation data sets.All the information of the data sets are summarized in **Table 1** and **Table S1**.

2. Microarray data processing

In order to merge the microarray data sets measured with 4 different platform chips (Agilent Whole Human Genome Microarray, Affymetrix HG-U133Plus2.0, HG-U133A, Illumina Human-6 v2), we selected genes from all platforms based on the NIH Entrez Gene ID and used the median rank score method with the R package CONOR[[1](#_ENREF_1)]for cross-platform normalization (**Figure S1**). Of 22,277 probes that were common among multiple platforms, 20,184 probes were selected to be in one to one relation between probe and gene. Coefficient of correlation (*r*) in each gene among microarray platforms was measured for considering the differences in microarray platforms. We evaluated the median value of each gene in both platforms and selected the gene sets with high correlation in which the absolute value of the extracting median values between 2 platforms was less than 1. From the resulting 8,920 genes, we removed genes that were not flagged as “detected” in more than 90% of training data set samples (n=273), considering them to have had either missing or uncertain expression signals. Moreover, all data were normalized per gene in each data set by log_2_ transforming the expression of each gene.

3. Weighted Gene Co-Expression Network

We used the R package “WGCNA” for network construction. To construct ECs-specific gene co-expression network, we followed the tutorials from the WGCNA website (<http://www.genetics.ucla.edu/labs/horvat/CoexpressionNetwork/Rpackages/WGCNA/Tu​torials/index.html>). A weighted gene co-expression network reconstruction algorithm was used to reconstruct co-expression networks for ECs. In addition, pathways (modules) and centrally located genes (module centroids) were also identified in WGCNA. Briefly, the WGCNA algorithm can be divided several parts. First, a gene co-expression network was constructed using gene expression profiles. Second, the gene modules were identified. Finally, the hub genes were found in interesting modules. The followings were the detailed procedure.

**3.1 Reconstruction of Gene Co-Expression Network**

A weighted gene co-expression network is fully specified by its adjacency matrix $a_{ij}$, a symmetric $n \times n$ matrix with entries in [0, 1] whose component $a_{ij}$ encodes the network connection strength between nodes *i* and *j*. To define the weighted co-expression network, a co-expression similarity matrix $s_{ij}$ which defined as the absolute value of the correlation coefficient between the profiles of nodes *i* and *j* was calculated. Then, a weighted network adjacency matrix function $a_{ij}$can be transformed by co-expression similarity.

(3.2)

(3.1)

$$s_{ij}=\left| cor\left( x_{i},x_{j} \right) \right|$$

$a_{ij}=s_{ij}^{\beta}$, *β* ≥ 1

The adjacency matrix was constructed using a soft power adjacency function *β.* This parameters *β* of the power function was defined in such a way that the resulting co-expression network (adjacency matrix) satisfies approximate scale-free topology. To measure how well the network satisfied a scale-free topology, we used fitting index R^2^ of the linear model that regressed $\log\left( p(k) \right)$ on $\log\left( k \right)$, where *k* is connectivity and$p(k)$is the frequency distribution of connectivity [[2](#_ENREF_2)]. The fitting index of a perfect scale-free network is 1. For our datasets, we chose a power of 6, which resulted to an approximate scale-free topology network with the scale-free fitting index *R^2^* greater than 0.8.

Then we define an adjacency function AF as a matrix valued function that maps an $n\times n$ dimensional adjacency matrix $a_{ij}$ onto a new $n\times n$ dimensional network adjacency $A=AF(a_{ij})$. In the following, the adjacency matrix can be transformed by the topological overlap matrix (TOM)-based adjacency function ${AF}^{TOM}$to the corresponding topological overlap matrix, i.e.,

$${AF}^{TOM}(a_{ij}^{oringinal})=\frac{\sum_{l\neq i,j} a_{il}^{original}a_{l,j}^{original}+a_{ij}^{original}}{\min\left( \sum_{l\neq i} a_{il}^{original},\sum_{l\neq j} a_{jl}^{original} \right)-a_{ij}^{original}+1}$$

The TOM-based adjacency function ${AF}^{TOM}$ is particularly useful in biological networks [[3](#_ENREF_3)] to reflect the gene-gene relative inter-connectedness. The topological overlap measure can serve as a filter that decreases the effect of spurious or weak connections, and it can lead to more robust networks [[4](#_ENREF_4),[5](#_ENREF_5)]

(3.3)

**3.2 Identifying Gene Module**

In WGCNA, the modules are defined the groups of genes with high topological overlap. Here, we briefly mention an approach for defining network modules which is used in WGCNA. The adjacency matrix (which is a measure of node similarity) turns into a dissimilarity measure. The dissimilarity transformation *D* (A) is introduced, i.e.,

(3.4)

$$D_{ij}\left( A \right)=1-A_{ij}$$

Note that *D* (A) is not satisfying our definition of an adjacency matrix since its diagonal elements equal 0. Alternatively, one can use the topological overlap matrix to define the TOM-based dissimilarity measure

(3.5)

$$\mathrm{dissTopOverlap}_{ij}=D_{ij}\left( {AF}^{TOM}\left( A \right) \right)=1-{TopOverlap}_{ij}$$

$$=1-\frac{\sum_{u\neq i,j} A_{iu}A_{ui}+A_{ij}}{\min\left( \sum_{u\neq i} A_{iu},\sum_{u\neq j} A_{ju} \right)+1-A_{ij}}$$

Then, the gene co-expression networks are defined by applying the TOM-based dissimilarity ${dissTopOverlap}_{ij}$ to cluster gene expression profiles, as illustrated in **Figure 2**.

The WGCNA used TOM-based dissimilarity ${dissTopOverlap}_{ij}$ in conjunction with the average linkage hierarchical clustering model to group genes into modules. The gene modules correspond to branches of the hierarchical clustering tree (dendrogram). Then we choose a height cutoff (0.2) to cut branches off the tree to determine the gene modules. The resulting branches correspond to gene modules, and can be visualized and identified in TOM plot (**Figure S2**). Here the modules are found by inspection: a height cutoff value is chosen in the dendrogram such that some of the resulting branches correspond to dark squares (modules) along the diagonal of the TOM plot. To date, this module detection approach has led to biologically meaningful modules in previous studies [[2](#_ENREF_2),[6](#_ENREF_6),[7](#_ENREF_7)]

**3.3 Identifying Cancer Hub Genes**

Several studies have shown that the relationship between connectivity and node significance (i.e., the hub gene significance) carries important biological information. For example, in the study of yeast networks where nodes correspond to genes, highly connected hub genes are essential for yeast survival, and hub genes tends to be preserved across species [[8-10](#_ENREF_8)]. To screen the hub genes in the ECs-specific networks, we provided a systematic framework for integrating WGCNA and genetic data. The following steps summarized our overall approach:

1. Reconstruct the co-expression networks from large-scale gene expression data
2. Study the functional enrichment (gene ontology, etc.) of network modules
3. Link module to the phenotypic characteristics of ECs, finding ECs-specific co-expression network.
4. Calculate the scaled connectivity ($K_{i}$) and gene significance (${GS}_{i}$) of networks for screening the potential hub genes.
5. Identify key hub genes regulating modules and phenotypic characteristics of ECs using elastic-net analysis.

**3.3.1 Potential Hub Genes**

As mention before, we have reconstructed the ECs-specific co-expression networks by WGCNA procedure. In Step 4, we identify potential hub genes by evaluating the genes with highly scaled connectivity and significantly gene significance in the ECs-specific co-expression networks. To measure the association between connectivity and gene significance, the WGCNA proposed the following measure of *hub gene significance*:

$$HubGeneSignif=\frac{\sum_{i} {GS}_{i}K_{i}}{\sum_{i} \left( K_{i} \right)^{2}},$$

Where the *GS* is a gene significance measure and *Ki* is the scaled connectivity. In the thesis, the *GS* is defined a trait-based gene significance measure as the absolute correlation between the the *i*-th gene expression profile *x_i_* and the sample trait *T*:

$${GS}_{i}=\left| cor\left( x_{i},T \right). \right|$$

The scaled connectivity *K*_i_ of the *i*th gene is defined by

$${ScaledConnectivity}_{i}=\frac{k_{i}}{k_{max}}=K_{i}$$

Where $k_{i}$ is the connectivity of gene, $k_{max}$ is the maximum connectivity of the gene. By definition, $0\leq K\leq1$.When *GS*_i_ is proportional to the scaled connectivity (${GS}_{i}=K_{I}$), the hub gene significance equals the constant of proportionality: ${GS}_{i}=c$. The hub gene significance equals the slope of the regression line between ${GS}_{i}$ and $K_{i}$ if the intercept term is set to 0. Only genes present in the value of GS and K more than 0.2 were selected as potential hub genes.

**3.3.2 Elastic-net analysis**

In the step 5, we take the previously described approach of the elastic net, a multivariate variable selection technique with a penalization approach. Potential hub genes were used as input variables. The penalized logistic regression models via the elastic net were used to select the key hub genes.

The elastic net analysis was used to select which of these features were associated with phenotypic characteristics of ECs across all data sets.

Let X be a $n \times p$ matrix of input features (where p is the number of features and n is the number of samples) and y be a vector of phenotypic characteristics of length n. For any non-negative *λ*_1_ and *λ*_2_

$L\left( \lambda_{1}, \lambda_{2},\beta\right)=\left| y-X\beta\right|^{2}+\lambda_{2}\sum_{j=1}^{P} \beta_{j}^{2}+\lambda_{1}\sum_{j=1}^{P} \left| \beta_{j} \right|$ (5.1)

Let $\hat{\beta}$ be the naive elastic net estimator. Then $\hat{\beta}=argmin\left\{ L\left( \lambda_{1}, \lambda_{2},\beta\right) \right\}$. A scaling factor of (1+*λ*_2_) is added to the naïve elastic net to prevent double shrinking.

$\hat{\beta}\left( \mathrm{elasticnet} \right)=(1+\lambda_{2})\hat{\beta}(naiveelasticnet)$ (5.2)

To determine the optimal $\lambda_{1}$ and $\lambda_{2}$, we let $\alpha=\lambda_{2}/(\lambda_{1}+\lambda_{2})$. Then $(1-\alpha)\sum_{j=1}^{P} \beta_{j}^{2}+\alpha\sum_{j=1}^{P} \left| \beta_{j} \right|$ is the elastic net penalty. Tenfold cross validation is performed to optimize $\lambda_{1}$ and $\lambda_{2}$ in equation (5.2), denoted as $\hat{\lambda}_{1}$ and $\hat{\lambda}_{2}$, respectively. To find the variables that associated with phenotypic characteristics of ECs, $\hat{\lambda}_{1}$, $\hat{\lambda}_{2}$, X and y are inputted into equation (5.2) to solve for vector *β*. In this study, we use a library 'glmnet' in R statistical package http://www.r-project.org website to conduct the elastic-net regression analysis.

This procedure was repeated 1000 times for each phenotype of ECs to assess the stability of the when applying the tenfold cross validation procedure. For each the 100 runs, a feature list was built for the phenotype comprised of genes. Features with higher stability of correlation in cross- validation (*f*) were considered of the highest confidence of truly being associated with phenotypic characteristics of ECs. Only potential hub genes present in more than fourth quartile (*f >*750) of all bootstrap samples were selected as ECs-specific hub genes. The results were shown in **Table 3**.

**3.4 Classifier model**

Here, we used the penalized logistic regression via the elastic net as the classifier model to predict the phenotypic characteristics of ECs. To build a classifier model, we assigned the phenotypic characteristics of ECs to classes, including high grade (G3, poorly differentiated) versus low grade (G1, well differentiated), Type I (estrogen dependent) versus Type II (estrogen independent), and later stage (higher than stage 2) versus early stage (lower than stage 2). The model is described as followings.

$$logit\left( p \right)=\beta_{0}+\sum_{i=1}^{N} \beta_{1}g_{i}$$

Where *p* represents the probability of getting the case (high grade, Type II or later stage ECs), and *g_i_*is the *i*^th^ gene in a given gene co-expression network.

Classification performance was assessed with areas under the receiving operating characteristic (AUC) curve. Using the training dataset, a classification model was built, and its discriminatory capacity was first estimated with 10-fold cross-validation (**Figure S3**). The resulting model was next tested on independent datasets using the key hub genes as a model input to predict the classes of particular samples relevant to the process of neoplastic transformation and progression in ECs (**Figure 5**).

**Reference**

1. Rudy J, Valafar F (2011) Empirical comparison of cross-platform normalization methods for gene expression data. Bmc Bioinformatics 12: 467.

2. Zhang B, Horvath S (2005) A general framework for weighted gene co-expression network analysis. Stat Appl Genet Mol Biol 4: Article17.

3. Ravasz E, Somera AL, Mongru DA, Oltvai ZN, Barabasi AL (2002) Hierarchical organization of modularity in metabolic networks. Science 297: 1551-1555.

4. Li A, Horvath S (2007) Network neighborhood analysis with the multi-node topological overlap measure. Bioinformatics 23: 222-231.

5. Yip AM, Horvath S (2007) Gene network interconnectedness and the generalized topological overlap measure. Bmc Bioinformatics 8: 22.

6. Horvath S, Zhang B, Carlson M, Lu KV, Zhu S, et al. (2006) Analysis of oncogenic signaling networks in glioblastoma identifies ASPM as a molecular target. Proceedings of the National Academy of Sciences of the United States of America 103: 17402-17407.

7. Carter SL, Brechbuhler CM, Griffin M, Bond AT (2004) Gene co-expression network topology provides a framework for molecular characterization of cellular state. Bioinformatics 20: 2242-2250.

8. Albert R, Barabasi AL (2000) Topology of evolving networks: local events and universality. Physical review letters 85: 5234-5237.

9. Oldham MC, Horvath S, Geschwind DH (2006) Conservation and evolution of gene coexpression networks in human and chimpanzee brains. Proceedings of the National Academy of Sciences of the United States of America 103: 17973-17978.

10. Han JD, Bertin N, Hao T, Goldberg DS, Berriz GF, et al. (2004) Evidence for dynamically organized modularity in the yeast protein-protein interaction network. Nature 430: 88-93.
