# Supplementary figures and images for: Visual gene-network analysis reveals the cancer gene co-expression in human endometrial cancer
Source: BMC Genomics. 2014 Apr 23;15:300. doi: 10.1186/1471-2164-15-300 (PMC4234489; doi:10.1186/1471-2164-15-300)

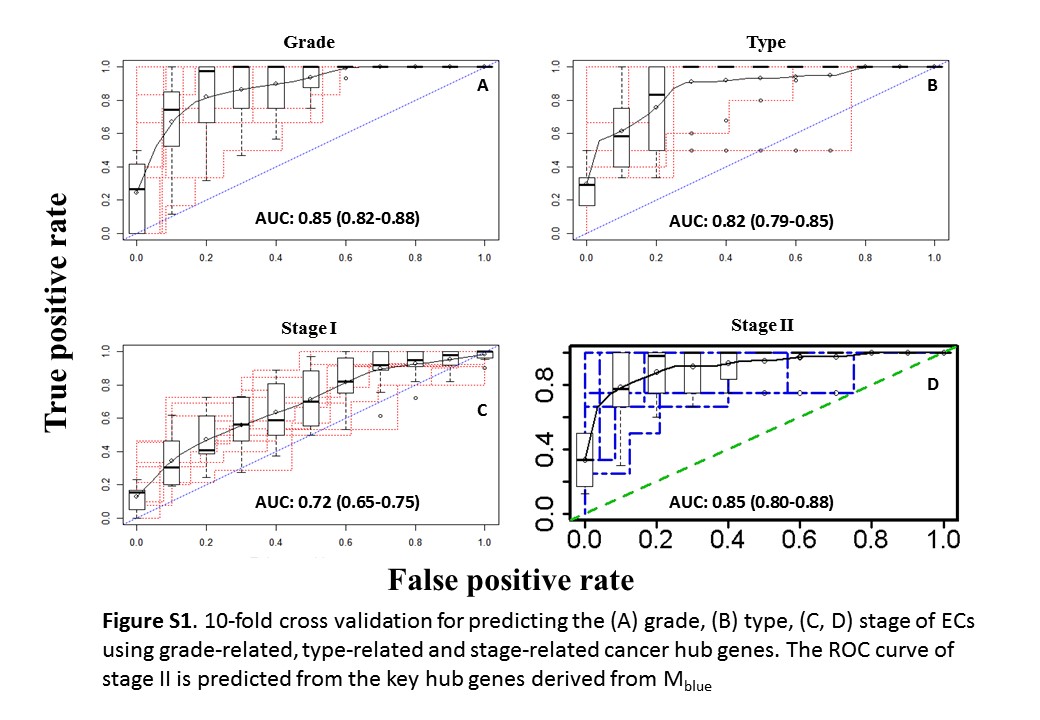

Supplement: Additional file 4: Figure S1 — 10-fold cross validation to predict the (A) grade, (B) type, (C, D) stage of ECs using grade-, type- and stage-related cancer hub genes. The ROC curve of stage II is predicted from the cancer hub genes derived from Mblue. [file 1471-2164-15-300-S4.jpg]

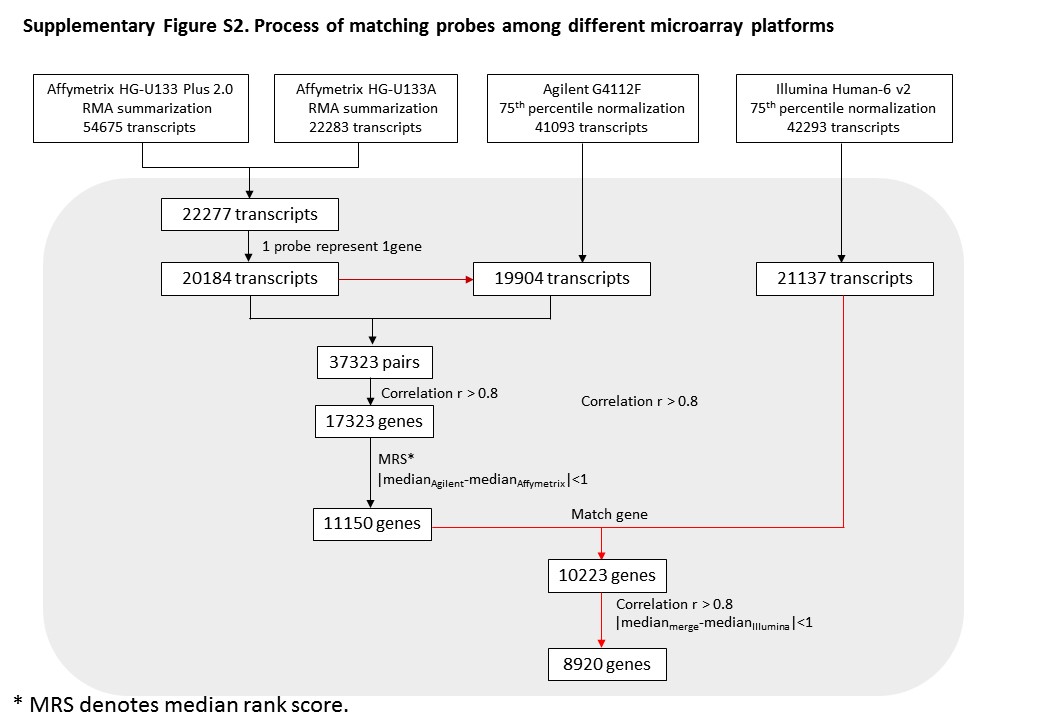

Supplement: Additional file 6: Figure S2 — Detailed process of matching probes among different microarray platforms. [file 1471-2164-15-300-S6.jpg]

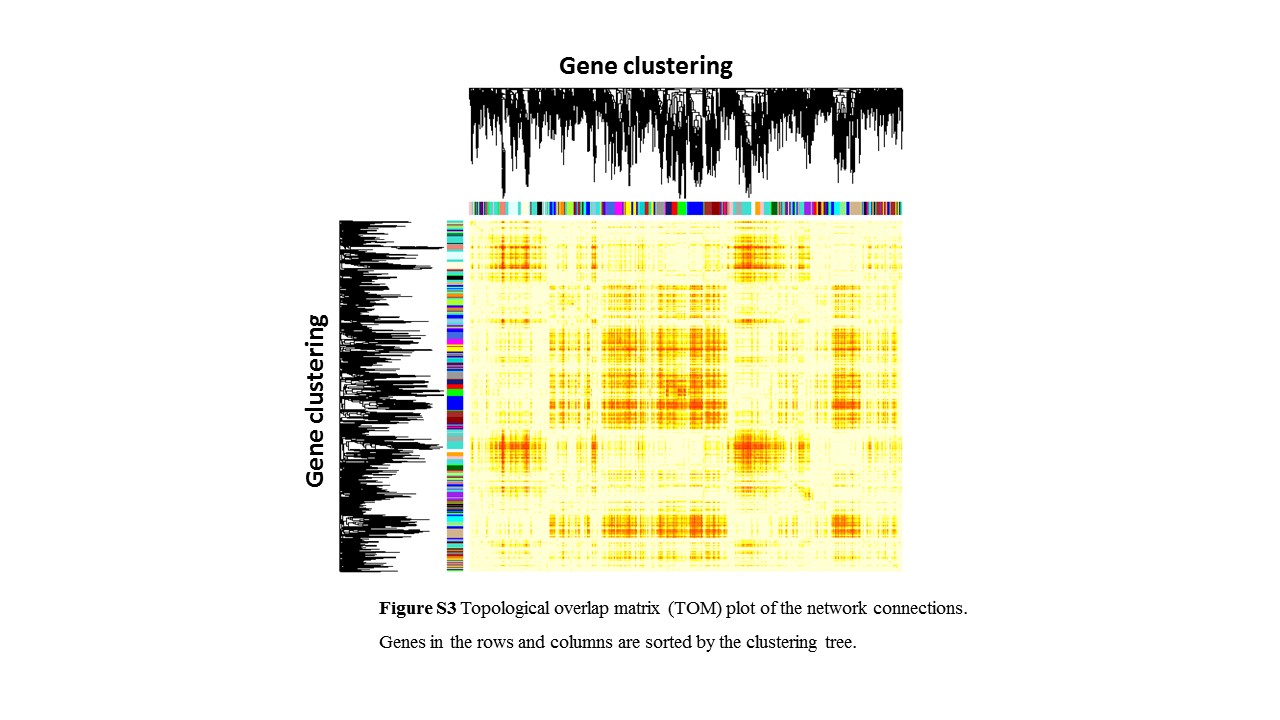

Supplement: Additional file 7: Figure S3 — Topological overlap matrix (TOM) plot of the network connections. Genes in the rows and columns are sorted by the clustering tree. [file 1471-2164-15-300-S7.jpg]
