# Supplementary material for: Visual gene-network analysis reveals the cancer gene co-expression in human endometrial cancer
Source: BMC Genomics. 2014 Apr 23;15:300. doi: 10.1186/1471-2164-15-300 (PMC4234489; doi:10.1186/1471-2164-15-300)
Supplement: Additional file 5: Table S3 — List of microarray experiments used for meta-analysis. [file 1471-2164-15-300-S5.docx]

**Table S3** Microarray experiments used for meta-analysis.

| **Array Express accession** | **Data set**^a^ | **Array no.** | **Array type**^b^ | **Probe length (nt)** | **Label** | **Signal type** | **Reference** |
| --- | --- | --- | --- | --- | --- | --- | --- |
| E-GEOD-17025 | Training set | 103 | I | 25 | in situ oligonucleotide | raw | [[1](#_ENREF_1)] |
| E-GEOD-6364 | Training set | 37 | I | 25 | in situ oligonucleotide | raw | [[2](#_ENREF_2)] |
| E-MTAB-1007 | Training set | 76 | II | 60 | in situ oligonucleotide | raw | [[3](#_ENREF_3)] |
| E-GEOD-33723 | Training set | 38 | II | 60 | in situ oligonucleotide | raw | [[4](#_ENREF_4)] |
| E-GEOD-23339 | Training set | 19 | III | 50 | oligonucleotide beads | raw | [[5](#_ENREF_5)] |
| E-GEOD-23518 | Validation set | 40 | III | 50 | oligonucleotide beads | raw | [[6](#_ENREF_6)] |
| E-GEOD-21882 | Validation set | 90 | IV | 69 | spotted oligonucleotide | raw | [[7](#_ENREF_7)] |

^a^Datasets are assigned to training sets or validation sets for evaluating predictive model.

^b^Microarrayplatform: I, AffymetrixGeneChip Human Genome U133 Plus 2.0 [HG-U133_Plus_2]; II, A-AGIL-28 - Agilent Whole Human Genome Microarray 4x44K 014850 G4112F (85 cols x 532 rows); III, A-MEXP-930 - Illumina Human-6 v2 Expression BeadChip; IV, A-GEOD-10422 - SWEGENE H_v3.0.1;

**References**

1. Day RS, McDade KK, Chandran UR, Lisovich A, Conrads TP, et al. (2011) Identifier mapping performance for integrating transcriptomics and proteomics experimental results. BMC Bioinformatics 12: 213.

2. Burney RO, Talbi S, Hamilton AE, Vo KC, Nyegaard M, et al. (2007) Gene expression analysis of endometrium reveals progesterone resistance and candidate susceptibility genes in women with endometriosis. Endocrinology 148: 3814-3826.

3. Mannelqvist M, Stefansson IM, Wik E, Kusonmano K, Raeder MB, et al. (2012) Lipocalin 2 expression is associated with aggressive features of endometrial cancer. Bmc Cancer 12.

4. Montserrat N, Mozos A, Llobet D, Dolcet X, Pons C, et al. (2012) Epithelial to mesenchymal transition in early stage endometrioid endometrial carcinoma. Human Pathology 43: 632-643.

5. Hawkins SM, Creighton CJ, Han DY, Zariff A, Anderson ML, et al. (2011) Functional MicroRNA Involved in Endometriosis. Molecular Endocrinology 25: 821-832.

6. Mhawech-Fauceglia P, Wang D, Kesterson J, Clark K, Monhollen L, et al. (2010) Microarray Analysis Reveals Distinct Gene Expression Profiles Among Different Tumor Histology, Stage and Disease Outcomes in Endometrial Adenocarcinoma. Plos One 5.

7. Levan K, Partheen K, Osterberg L, Olsson B, Delle U, et al. (2010) Identification of a Gene Expression Signature for Survival Prediction in Type I Endometrial Carcinoma. Gene Expression 14: 361-370.
